# A prospective study on the efficacy of sequential treatment of technology Lipido‐Colloid Impregnated with Silver and Technology Lipido‐Colloid Nano‐Oligosaccharide Factor in the management of venous leg ulcers

**DOI:** 10.1002/hsr2.1488

**Published:** 2023-08-23

**Authors:** Natalie Shi Qi Wong, Audrey Hui Min Tan, Kai Siang Chan, Karine C. C. Goh, Peiting Lai, Sivakami Muthuveerappa, Mohamed Maliki Bin Mohamed Nasir, Shanying Liang, Qiantai Hong, Enming Yong, Zhiwen Joseph Lo

**Affiliations:** ^1^ Yong Loo Lin School of Medicine National University of Singapore Singapore Singapore; ^2^ Wound and Stoma Care, Nursing Specialty Tan Tock Seng Hospital Singapore Singapore; ^3^ Department of General Surgery Vascular Surgery Service, Tan Tock Seng Hospital Singapore Singapore; ^4^ Department of Surgery, Vascular Surgery Woodlands Health Singapore Singapore; ^5^ Lee Kong Chian School of Medicine Nanyang Technological University Singapore Singapore; ^6^ Skin Research Institute of Singapore Agency for Science Technology and Research Singapore Singapore

**Keywords:** chronic wounds, compression bandages, venous leg ulcer, wound dressing, wound healing

## Abstract

**Background and Aims:**

Venous leg ulcers (VLUs) are associated with significant morbidity and poor quality of life (QOL). Compression therapy and wound dressing are the mainstay treatment options. Technology Lipido‐Colloid Impregnated with Silver (TLC‐Ag) reduces bacterial load and Technology Lipido‐Colloid Nano‐Oligosaccharide Factor (TLC‐NOSF) reduces elevated matrix metalloproteinases and improve wound healing. However, evidence is scarce on the role of sequential therapy. This study aims to evaluate if sequential treatment with TLC‐Ag and TLC‐NOSF improves VLU wound healing and QOL.

**Methods:**

This is a prospective cohort study from May 2020 to October 2021 on patients with VLUs who received sequential therapy, consisting of 2 weeks of TLC‐Ag followed by two‐layer compression bandage (2LB) with TLC‐NOSF until complete wound healing. Participants were followed‐up with weekly dressing changes. Our primary outcomes were wound area reduction (WAR) and Pressure Ulcer Scale of Healing (PUSH) score. Our secondary outcomes were QOL measures.

**Results:**

There were 28 patients with 57.1% males (*n* = 16) with a mean age of 65.3 years. Mean duration of VLU was 13.9 ± 11.7 weeks before the initiation of sequential therapy. Mean baseline wound area was 8.44 cm^2^. Median time to wound healing was 10 weeks. 57.1% of patients achieved complete wound closure at 3 months. There was significant WAR after 1 month (mean area 8.44–5.81 cm^2^, 31.2% decrease) and after 3 months (mean area 8.44–2.53 cm^2^, 70.0% decrease). Mean monthly WAR was 28.9%. PUSH score also decreased at 1 month (16.5% decrease, *p* < 0.001) and 3 months (63.3% decrease, *p* < 0.001) marks following the sequential therapy. EuroQol Visual Analog Scale (EQ‐VAS) improved following sequential therapy (baseline: 69.0 ± 15.0, week 13: 80.2 ± 13.2, *p* < 0.001).

**Conclusion:**

Sequential therapy with TLC‐Ag followed by TLC‐NOSF and 2LB is feasible, with good wound healing and improvement in QOL of patients with VLUs.

## Key points

- Technology Lipido‐Colloid Impregnated with Silver (TLC‐Ag) and Technology Lipido‐Colloid Nano‐Oligosaccharide Factor (TLC‐NOSF) have been shown to reduce bacterial load and improve wound healing. However, evidence is scarce on the use of sequential therapy with various wound dressings and compression therapy for patients with venous leg ulcers (VLUs).
- Our study aims to evaluate the efficacy and safety of a novel sequential therapy with 2 weeks of TLC‐Ag followed by TLC‐NOSF with two‐layer compression bandage for patients with VLUs.
- There was significant 1‐month (31.2%) and 3‐month (70.0%) wound area reduction with majority obtaining complete wound closure (57.1%) at 3 months. There was also significant improvement in quality of life quantified by the EuroQol Visual Analog Scale.

## 1 INTRODUCTION

Chronic venous insufficiency (CVI) is a chronic and debilitating disease with a prevalence of 25%–40% in females and 10%–20% in males.^1^, ^2^ Severe CVI may be complicated by venous leg ulcers (VLUs) or malignant transformation into Marjolin's ulcer.^3^, ^4^ VLUs are the most common type of chronic ulcers occurring in the lower extremities, prevalent in 1% of the global population.^5^ VLUs are associated with high chronicity, rates of recurrence, and poor quality of life (QOL). Chronic nonhealing wounds also predispose to secondary infections and further complications, resulting in higher morbidity.^6^ These inadvertent complications of VLUs, therefore result in significant socioeconomic burden^7^; locally in Singapore, 1‐year VLU recurrence was 52.5% with median interval of 9.5 months between healing and recurrence, with an estimated cost of US dollar (USD) 16,761 per patient.^7^

Current literature and standard clinical practice recommends a multimodal approach for the treatment of VLUs, involving compression therapy and wound care.^8^ Compression therapy has been shown to result in 60%–70% complete ulcer healing after 12–24 weeks of use.^9^ Another facet of management of VLU involves wound care, which consists of wound assessment, monitoring, and dressings.^10^ Recent evidence has shown that a two‐layer compression bandage (2LB) has similar time to healing compared to a four‐layer compression bandage (4LB), but with better cost‐effectiveness and associated with greater patient compliance.^11^, ^12^ Use of Technology Lipido‐Colloid with silver dressing (TLC‐Ag) has been shown to reduce bacterial load and wound area reduction (WAR).^13^ Technology Lipido‐Colloid Nano‐Oligosaccharide Factor (TLC‐NOSF) has also been shown to reduce the inflammatory cascade and improve WAR in VLUs.^14^, ^15^

A small noncomparative study on 36 patients showed use of UrgoTul® (Laboratoires URGO) and K‐Four® (Laboratoires URGO, Paris, France) showed that 50% of patients had healed VLU in 46.8 days following treatment and reported reduced pain at dressing change.^16^ However, there is a paucity of evidence on combination of compression therapy and wound dressing on VLU outcomes. A review in 2013 identified that while patients undergo sequential treatments, that is, application of different dressings over time, there is no evidence on the order or duration of each therapy.^17^ Clinical practice guidelines by the Society for Vascular Surgery similarly did not make any recommendations on the above.^8^ Hence, we propose a sequential protocol consisting of initial TLC‐Ag followed by combination of TLC‐NOSF with 2LB. Our aim is to evaluate the efficacy of this sequential protocol on healing of VLUs and QOL of patients.

## 2 MATERIALS AND METHODS

**Figure 1:**
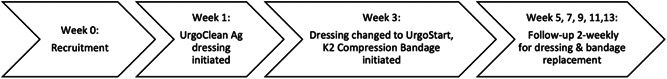
Study protocol outlining the timeline from recruitment to completion of study.

This is a noncomparative prospective single‐center study conducted at a 1300‐bed university‐affiliated tertiary hospital in Singapore. This is a 13‐week study (Figure 1) with a recruitment period from May 2020 to October 2021, and a study period from August 2020 to December 2021. Patients were recruited at Week 0, with treatment commenced at Week 1 and follow‐up completed at Week 13. The 12‐week follow‐up timeframe was adopted as compression therapy has been shown to result in 60%–70% complete ulcer healing after 12–24 weeks of use.^9^ Inclusion criteria were patients ≥21 years old, a VLU surface area between 2 and 50 cm^2^ lasting 1–12 months before recruitment and a normal ankle circumference and ankle‐brachial pressure index (ABPI). For patients with multiple ulcers, only one target ulcer which best met the selection criteria was chosen for evaluation. The other ulcers were treated with standard treatment procedures, which usually include silver‐based foam dressing together with compression therapy. Exclusion criteria were non‐VLUs or patients planned for venous surgery within the study period. The complete list of inclusion and exclusion criteria are outlined in Table 1. A total of 45 patients were recruited for the study; however, 17 patients were lost to follow‐up before completion of the study, leaving 28 patients which were included in the final analysis. This study was approved by the institutional ethics review board (National Healthcare Group Domain Specific Review Board Ref No: 2020/00466). The conduct of this study is in accordance with the CONSORT (CONsolidated Standards Of Reporting Trials) guidelines.^18^

**Table 1:** Inclusion and exclusion criteria used in our study.

Inclusion criteria	Exclusion criteria
Patient factors	Patient demographics/comorbidities
Age ≥ 21 years, male or female	
Able to provide written informed consent	On immunosuppressive drugs or high‐dose corticosteroids
Can be followed up by the same investigating team for the 13‐week treatment period	History of deep or superficial vein thrombosis 3 months prior to inclusion
Agrees to adhere to study protocol with respect to the type of multilayer compression system and primary wound dressing	Participation in another interventional clinical trial
Ankle circumference between 18 and 32 cm	Known hypersensitivity to one of the components in the compression bandage or wound dressing
	Venous surgery scheduled within 13‐week treatment period
Ulcer factors	Ulcer characteristics
Ulcer area 2–50 cm^2^	Partially or completely covered by black necrotic plaque
Ulcer duration 1–12 months	Ischemic ulcer (ABPI < 0.6)
ABPI 0.8–1.3 in both legs (UrgoK2)	Ulcer with eschar
ABPI 0.6<0.8 in both legs (UrgoK2 Lite)	Malignant ulcer
Study ulcer ≤3cm from any edge to another wound on the same limb	

Abbreviation: ABPI, ankle‐brachial pressure index.

### 2.1 Study protocol

All patients of our wound care specialist outpatient clinic (SOC) were screened for eligibility for the study by our on‐site principal investigator or dedicated wound nurse according to the inclusion and exclusion criteria. Patients who had received prior local treatment for VLUs were not excluded from the selection process. Eligible participants were briefed at a dedicated consultation room and opportunities were given to ask on the details of the study. An on‐site translator was available for non‐English participants. Informed consent was taken from each participant who agreed to enroll into the study. Patients that completed the study protocol were remunerated at the end of the study for their participation.

The study protocol is illustrated in Figure 1. Baseline patient and wound characteristics were collected from all participants at the start of the study. Each consultation subsequently was conducted at our wound care SOC. Following data collection, the VLUs were cleansed with normal saline solution. VLUs of enrolled subjects were dressed with UrgoClean Ag® (TLC‐Ag) (Laboratoires URGO) for 2 weeks, followed by 11 weeks of a combination of UrgoK2® or UrgoK2 Lite® (2LB) (Laboratoires URGO) together with UrgoStart® (TLC‐NOSF) (Laboratoires URGO). Neutral absorbent dressings were used for excess exudate management. Frequency of dressing and bandage changes was standardized to once per week but subject to clinical judgment. Use of additional local treatment such as local antiseptic pastes, zinc pastes, or corticosteroids were permitted for application around the wound. Such use of additional treatment were documented and recorded. All dressings and wound treatments were performed by our trained wound clinicians. Intermediate assessments were performed at 2‐weekly intervals where data was collected at every time point. Patients were recruited on Weeks 0 and 1 is defined as the week when participants begun the sequential treatment (hence resulting in a 12‐week follow‐up). Time intervals of “after 1 month” and “after 3 months” were, therefore, defined as Weeks 5 and 13, respectively. Data collection during the intermediate assessments was conducted by one dedicated research coordinator as much as possible for each review, and there was a total of three research coordinators. A deidentified digital image of the wound of the patient was taken using a white‐listed hospital‐approved camera (Canon G7X) during each visit, without any identifiers on the image. Patients were followed up until the end of the stipulated study period or until the wound has healed.

### 2.2 Study variables and outcomes

Baseline demographics including age, gender, comorbidities, and ulcer characteristics were recorded. Ulcer assessment included the Falanga wound bed score and Pressure Ulcer Scale of Healing (PUSH) score.^19^ The PUSH score is an easy‐to‐use scoring system which permits monitoring of global healing results in wound management due to its comprehensive description of wound exudate, size, and tissue type. While it was initially designed for characterizing pressure ulcers, its use has since been validated to be effective in the grading of VLUs.^20^, ^21^ The EuroQol 5‐dimensional 5‐level (EQ‐5D‐5L)^22^ was administered to each participant during the first (on Week 0) and last visit (on Week 13). Its use has been validated in monitoring the healing process of VLUs and discriminating between healed and nonhealed VLUs.^23^ The EQ‐5D‐5L consists of five domains: mobility, self‐care, usual activities, pain and discomfort, and anxiety and depression. Each domain is graded using a 5‐point Likert scale. 1 corresponds to *no complications*, and 5 corresponds to *severe complications*. Participants are represented by a five‐digit health state, 11111 being the best health state to 55555 being the worst, with a total of 3125 possible health states. From each health state, a single index utility score can be calculated using country‐specific value sets. For the purpose of this study, the crosswalk US value sets were used as a value set for Singapore has yet to be published on the EuroQol database.^22^ The questionnaire also includes a visual analog scale (EQ‐VAS) as a global rating of self‐perceived health, which has a maximum score of 100 indicating “the best health you can imagine.” Our primary outcomes were incidence of complete wound closure, WAR and PUSH score. A healed VLU was defined as complete wound closure. Our secondary outcomes were health‐related QOL measures, that is, incidence of perfect health state (11111) measured using the EQ‐5D‐5L, utility score (calculated using the country‐specific value sets), and EQ‐VAS score.

### 2.3 Statistical analysis

All statistical analyses were performed using Jamovi Version 2.3.18 (Jamovi).^24^ Categorical variables were expressed as *n* (%), and continuous variables were described as mean ± standard deviation (SD) unless otherwise specified. Two‐sided paired samples *t* test was used to compare VLU wound size and QOL outcomes before and after implementation of the treatment protocol. Statistical significance was defined with an a priori level of significance set as *p* < 0.05. Kaplan–Meier analysis was used to analyze the cumulative time to wound healing.

## 3 RESULTS

### 3.1 Patient demographics

Patient demographics are detailed in Table 2. There were 28 patients included in the final analysis. Majority of participants were males (*n* = 16/28, 57.1%) with a mean age of 65.3 years. Most patients were Chinese (57.1%, *n* = 16/28). Majority of the participants had pre‐existing cardiovascular comorbidities: 53.6% (*n* = 15/28) had diabetes mellitus, 67.9% (*n* = 19/28) had hypertension, and 71.4% (*n* = 20/28) had peripheral vascular disease. With regard to the history on CVI, there were 46.4% (*n* = 13/28) with varicose veins, 75% (*n* = 21/28) with previous venous ulcers, 38.3% (*n* = 11/28) with a history of venous surgery and 7.1% (*n* = 2/28) with a history of deep vein thrombosis. Laterality of VLU was similar; 46.4% were right‐sided (*n* = 13/28) and 53.6% were left‐sided (*n* = 15/28). The mean duration of the VLUs was 13.9 ± 11.65 weeks before the sequential treatment. The mean PUSH score was 11.0 ± 2.25 with a lowest score of 7 and a highest score of 15 at the start of the study (Table 3).

**Table 2:** Patient demographics (*n* = 28 patients).

Demographics	Overall, *n* (%)
Age, mean (SD)	65.3 (13.8)
Gender = Male (%)	16/28 (57.1)
Ethnicity	
Chinese (%)	16/28 (57.1)
Malay (%)	5/28 (17.9)
Indian (%)	2/28 (7.1)
Others (%)	5/28 (17.9)
Comorbidities	
Diabetes mellitus (%)	15/28 (53.6)
Hypertension (%)	19/28 (67.9)
Peripheral vascular disease (%)	20/28 (71.4)
Characteristics of chronic venous insufficiency	
Presence of varicose veins (%)	13/28 (46.4)
Previous history of venous ulcers (%)	21/28 (75.0)
Previous venous surgery (%)	11/28 (39.3)
Previous deep vein thrombosis (%)	2/28 (7.1)

**Table 3:** Study characteristics of ulcers in all included patients (*n* = 28).

Baseline characteristics	*n* (%)
Laterality = Right (%)	13/28 (46.4%)
Duration of current leg ulcer in weeks, mean (SD)	13.9 (11.65)
PUSH score, mean (SD)	11.0 (2.25)
Area of ulcer in cm^2^, mean (SD)	8.44 (7.41)
A4 = 1.1–2.0 (%)	2 (7.1)
A5 = 2.1–3.0 (%)	6 (21.4)
A6 = 3.1–4.0 (%)	3 (10.7)
A7 = 4.1–8.0 (%)	5 (17.9)
A8 = 8.1–12.0 (%)	4 (14.3)
A9 = 12.1–24.0 (%)	7 (25.0)
A10 ≥ 24.0 (%)	1 (3.6)
Exudate amount	
E0 = None (%)	1 (3.6)
E1 = Light (%)	11 (39.3)
E2 = Moderate (%)	15 (53.6)
E3 = Heavy (%)	1 (3.6)
Tissue type	
T1 = Epithelial tissue (%)	3 (10.7)
T2 = Granulation tissue (%)	12 (42.9)
T3 = Slough (%)	12 (42.9)
T4 = Necrotic tissue (%)	1 (3.6)

Abbreviation: PUSH, Pressure Ulcer Scale of Healing.

### 3.2 Primary outcomes

**Figure 2:**
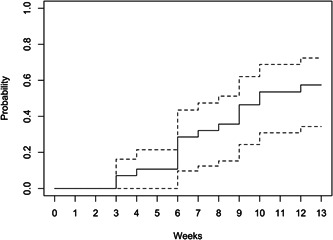
Kaplan–Meier analysis of cumulative wound healing over 13 weeks. Dotted lines = 95% confidence interval (CI).

**Figure 3:**
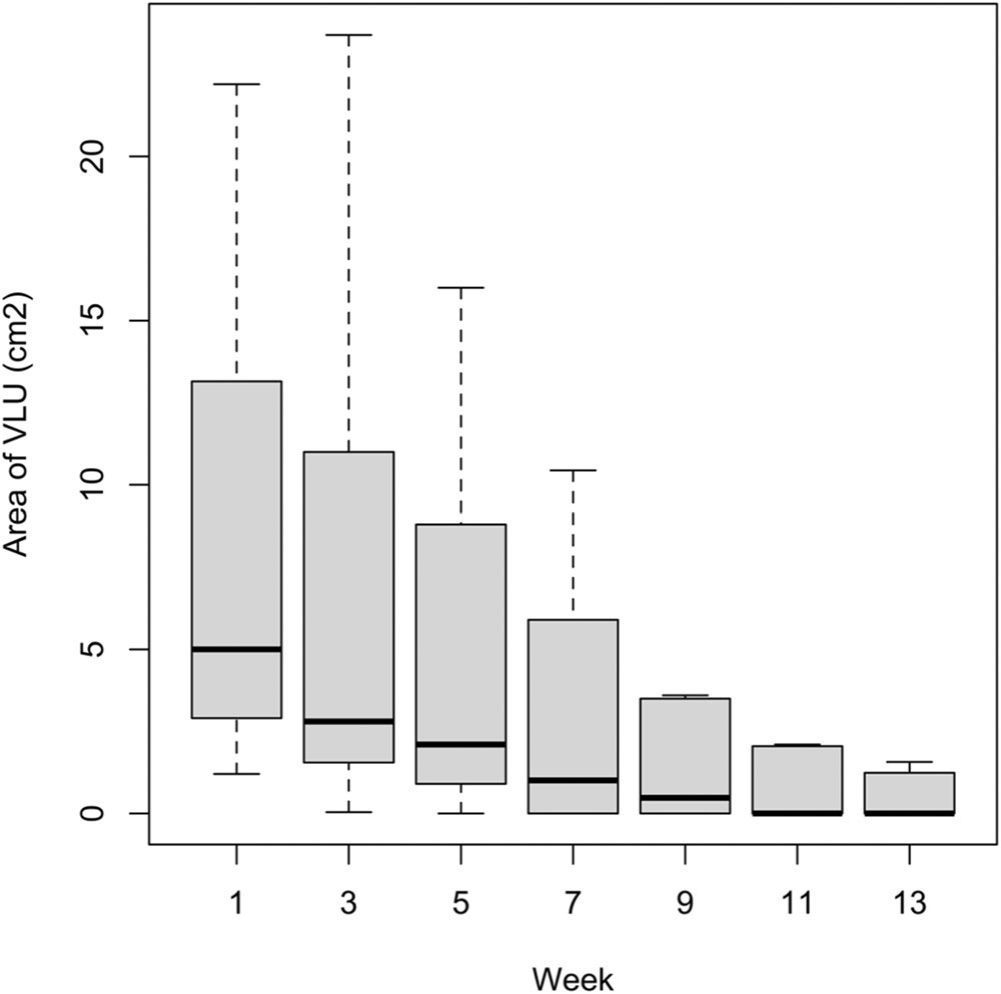
Area of venous leg ulcer (VLU) over time. Bar, interquartile range (IQR); horizontal line, median; vertical line, standard deviation (SD).

**Figure 4:**
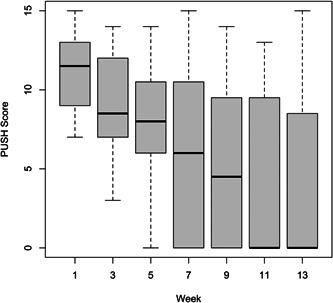
Pressure Ulcer Scale of Healing (PUSH) score of venous leg ulcer (VLU) over time. Bar, interquartile range (IQR); horizontal line, median; vertical line, standard deviation (SD).

Cumulative wound healing is illustrated in Figure 2 using the Kaplan–Meier analysis. Median time to wound healing was 10 weeks (95% confidence interval [CI] = 7.41, 12.6). Figures 3 and 4 illustrate the bar plots for the VLU areas and PUSH scores over 13 weeks, which similarly showed a decline in VLU wound area and PUSH scores at every 2‐week interval.

1‐ and 3‐month primary outcomes are described in Table 4. Out of 28 patients, 3 patients (10.6%, *p* = 0.08) achieved complete VLU wound closure in 1 month (Week 5). At the end of the study after 3 months (Week 13), majority of patients (*n* = 16/28, 57.1%) achieved complete closure. Incidence of complete wound closure was significantly higher at the 3‐month mark compared to 1‐month mark (571.% vs. 10.7%, *p* < 0.001).

**Table 4:** 1‐ and 3‐month outcomes after the treatment protocol.

Outcome	1 month (Week 5)	*p* Value	3 months (Week 13)	*p* Value
No. of patients with fully healed VLU (%)	3 (10.7%)	0.08	16 (57.1%)	**<0.001**
VLU area, mean (SD)	5.81 ± 9.14	**0.02**	2.53 ± 4.88	**<0.001**
PUSH score, mean (SD)	7.79 ± 3.95	**<0.001**	4.04 ± 5.26	**<0.001**
Area of ulcer in cm^2^, mean (SD)				
A0 = 0 (%)	3 (10.7)		16 (57.1)	
A1 ≤ 0.3 (%)	2 (7.1)		0 (0.0)	
A2 = 0.4–0.6 (%)	0 (0.0)		1 (3.6)	
A3 = 0.7–1.0 (%)	3 (10.7)		3 (10.7)	
A4 = 1.1–2.0 (%)	4 (14.3)		1 (3.6)	
A5 = 2.1–3.0 (%)	7 (25.0)		1 (3.6)	
A6 = 3.1–4.0 (%)	1 (3.6)		0 (0.0%	
A7 = 4.1–8.0 (%)	0 (0.0)		1 (3.6)	
A8 = 8.1–12.0 (%)	4 (14.3)		2 (7.1)	
A9 = 12.1–24.0 (%)	3 (10.7)		3 (10.7)	
A10 ≥ 24.0 (%)	1 (3.6)		0 (0.0)	
Exudate amount				
E0 = None (%)	4 (14.3)		16 (57.1)	
E1 = Light (%)	18 (64.3)		5 (17.9)	
E2 = Moderate (%)	5 (17.9)		7 (25.0)	
E3 = Heavy (%)	1 (3.6)		0 (0.0)	
Tissue type				
T0 = Closed (%)	3 (10.7)		16 (57.1)	
T1 = Epithelial tissue (%)	5 (17.9)		3 (10.7)	
T2 = Granulation tissue (%)	17 (60.7)		7 (25.0)	
T3 = Slough (%)	1 (3.6)		1 (3.6)	
T4 = Necrotic tissue (%)	2 (7.1)		1 (3.6)	

*Note*: Bold values indicate statistically significant as *p* < 0.05.

Abbreviations: PUSH, Pressure Ulcer Scale of Healing; VLU, venous leg ulcer.

There was significant decrease in mean VLU area after 1 month (decrease by 31.2%, Week 0: 8.44 ± 7.41 cm^2^; Week 5: 5.81 ± 9.14 cm^2^; *p* = 0.02) and after 3 months (decrease by 70.0%, Week 13: 2.53 ± 4.88 cm^2^; *p* < 0.001) compared to the start of the study. On average, the monthly VLU WAR was 28.9%; VLU WAR was 33.5% from the 1st to 2nd month, and the VLU WAR was 22.0% from the 2nd to 3rd months. There was also a significant decrease in PUSH score after 1 month (decrease by 16.5%, Week 0: 11.0 ± 2.25; Week 5: 7.79 ± 3.95; *p* < 0.001) and after 3 months (decrease by 63.3%, Week 13: 4.04 ± 5.26; *p* < 0.001) compared to start of the study.

Subgroup analysis of patients with healed VLUs found that the mean time to healing was 9.64 ± 3.54 weeks. The greatest number of patients achieving complete wound closure was observed at Week 6, with five additional patients (17.9%) compared to the previous week.

### 3.3 Secondary outcomes

Tables 5, 6, 7 illustrates QOL comparisons before and after the treatment protocol. In the overall cohort, there was a significant improvement in EQ‐VAS score after the sequential treatment protocol (Week 0: 69.0 ± 15.0; Week 13: 80.2 ± 13.2; *p* < 0.001). Incidence of perfect health states [Week 0: *n* = 3/28 (10.7%) vs. Week 13: *n* = 6/28 (21.4%), *p* = 0.33] and utility scores (Week 0: 0.792 ± 0.175; Week 13: 0.845 ± 0.177; *p* = 0.17) were comparable before and after the sequential treatment protocol.

**Table 5:** EuroQol 5‐dimensional 5‐level (EQ‐5D‐5L) health states, all versus healed versus nonhealed venous leg ulcers.

Health state	Before	After	*p* Value
All patients (*n* = 28)			0.33
Perfectly healthy (11111)	3 (10.7%)	6 (21.4%)	
Less than perfectly healthy (>11111)	25 (89.3%)	22 (78.6%)	
Healed (*n* = 16)			**0.04**
Perfectly healthy (11111)	0 (0%)	4 (25%)	
Less than perfectly healthy (>11111)	16 (100%)	12 (75%)	
Nonhealed (*n* = 12)			0.67
Perfectly healthy (11111)	3 (25%)	2 (16.7%)	
Less than perfectly healthy (>11111)	9 (75%)	10 (83.3%)	

*Note*: Bold value indicates statistically significant as *p* < 0.05.

**Table 6:** EuroQol 5‐dimensional 5‐level (EQ‐5D‐5L) domain‐specific scores, all versus healed versus nonhealed venous leg ulcers.

Domain, mean (SD)	All patients (*n* = 28)			Healed (*n* = 16)			Nonhealed (*n* = 12)		
	Before	After	*p* Value	Before	After	*p* Value	Before	After	*p* Value
Mobility	1.79 (0.876)	1.82 (0.945)	0.84	2.06 (0.929)	1.75 (0.931)	0.14	1.42 (0.669)	1.92 (0.996)	0.08
Self‐care	1.39 (0.685)	1.25 (0.645)	0.29	1.38 (0.619)	1.31 (0.793)	0.58	1.42 (0.793)	1.17 (0.389)	0.39
Usual activities	1.68 (0.863)	1.357 (0.731)	0.06	1.69 (0.946)	1.38 (0.806)	0.17	1.67 (0.778)	1.33 (0.651)	0.22
Pain/discomfort	2.18 (0.819)	1.79 (0.876)	0.07	2.25 (0.775)	1.63 (0.619)	**≤0.01**	2.08 (0.900)	2.00 (1.13)	0.85
Anxiety/depression	1.71 (0.854)	1.36 (0.731)	0.12	1.94 (0.772)	1.25 (0.577)	**0.02**	1.42 (0.900)	1.50 (0.905)	0.82

*Note*: Bold values indicate statistically significant as *p* < 0.05.

**Table 7:** Mean utility and EuroQol‐visual analog scales (EQ‐VAS) scores, all versus healed versus nonhealed venous leg ulcers.

Scores, mean (SD))	All patients (*n* = 28)			Healed (*n* = 16)			Nonhealed (*n* = 12)		
	Before	After	*p* Value	Before	After	*p* Value	Before	After	*p* Value
Utility	0.792 (0.175)	0.845 (0.177)	0.17	0.765 (0.162)	0.870 (0.169)	**≤0.01**	0.829 (0.191)	0.812 (0.189)	0.81
EQ‐VAS	69.0 (15.0)	80.2 (13.2)	<0.001*	66.8 (15.4)	81.3 (14.2)	**≤0.01**	72.1 (14.5)	78.7 (12.3)	0.05

*Note*: Bold values indicate statistically significant as *p* < 0.05.

Subgroup analysis of patients (*n* = 16) with healed VLUs after 12 weeks, however, showed significantly higher incidence of perfect health states [Week 0: *n* = 0; Week 13: *n* = 4/16 (25.0%); *p* = 0.04], higher utility scores (Week 0: 0.765 ± 0.162; Week 13: 0.870 ± 0.169; *p* = 0.005) and EQ‐VAS score (Week 0: 66.8 ± 15.4; 81.3 ± 14.2; *p* = 0.003). For patients who had nonhealed VLUs (*n* = 12), there was no improvement in domain‐specific scores of the EQ‐5D‐5L. While there were higher utility scores in healed VLUs compared to nonhealed VLUs, this was not statistically significant (healed: 0.870 ± 0.169 vs. nonhealed: 0.812 ± 0.189, *p* = 0.39).

## 4 DISCUSSION

The use of compression therapy has been validated and is recommended as mainstay therapy in treating VLUs.^8^, ^9^ However, evidence on the use of sequential therapy of different wound dressings in combination with compression therapy is scarce. Our study demonstrates the feasibility and efficacy of a sequential treatment protocol with 2 weeks of TLC‐Ag, followed by an 11‐week combination of TLC‐NOSF and 2LB.

In present guidelines, silver‐containing dressings, such as TLC‐Ag, are indicated for VLUs that are at risk of infection or showing clinical signs of local infection.^25^ An open‐label randomized controlled trial (RCT) in 2012 on 102 patients with heavily colonized VLUs demonstrated significant improvement in WAR with TLC‐Ag dressings compared to neutral TLC dressings in the initial 4 weeks (47.9% vs. 5.6%, *p* = 0.04).^13^ Heavily colonized wounds were defined as the presence of ≥3 of the following: pain between dressing changes, perilesional skin erythema, edema, foul odor, and heavy exudation.^13^ Translational study on the use of nanocrystalline silver dressing in VLU also showed significant reduction in bacterial count after 12 weeks (*p* = 0.01) and demonstrated that heavy neutrophil infiltration is associated with delayed wound healing.^25^ Hence, our study adopted the use of TLC‐Ag during the first 2 weeks of the treatment protocol as a bridging regimen to reduce bacterial load and promote wound healing for subsequent sequential therapy with combination 2LB and TLC‐NOSF.

The use of TLC‐NOSF in VLU care pathways is been recommended in the National Institute for Health and Care Excellence (NICE) guidelines.^26^ The efficacy of TLC‐NOSF has been demonstrated in chronic wounds of various etiologies, for example, pressure ulcers, diabetic foot ulcers, and VLUs.^27^ A double‐blinded RCT on 187 patients with VLU showed significantly higher WAR with TLC‐NOSF compared to neutral TLC (58.3% vs. 31.6%, *p* = 0.002) after 8 weeks of treatment.^14^ The pathophysiology of VLUs can be attributed to the inflammatory cascade. In CVI, increased hydrostatic pressure in the venous system results in shear stress on the endothelium resulting in inflammation.^28^ Expression of matrix metalloproteinases (MMPs) and cytokines during the inflammatory process results in damage to venous walls with eventual extension to the dermis, resulting in VLUs.^29^ MMP‐1 and MMP‐8 have also been demonstrated to be responsible for nonhealing VLUs.^30^, ^31^ Utilization of NOSF in TLC‐NOSF reduces MMPs and has been demonstrated to improve wound healing, with mean time‐to‐wound closure reported to be 58 ± 24 days in the NEREIDES study (inclusion criteria were wounds with ≥70% sloughy tissue) and 55 ± 23 days in the CASSIOPEE study (inclusion criteria were wounds with ≥50% granulation tissue), where cohorts of patients with VLU or mixed ulcers were studied.^32^ Our sequential therapy similarly showed promising results with mean time to wound closure of 67.5 days.

While TLC‐NOSF was initiated as the second part of our sequential therapy, it was only initiated after a short 2 weeks of TLC‐Ag. A pooled data analysis of 10,220 patients with chronic wounds showed that the shortest time‐to‐closure was reached when wounds were treated with first‐line TLC‐NOSF dressings (70.2 vs. 103.7 days, *p* < 0.001) regardless of severity and nature of chronic wound.^27^ They defined second‐line as follow‐up for chronic wounds and not previously treated with TLC‐NOSF, but did not specify the type and duration of the initial dressing given. While we initiated TLC‐NOSF following dressing with TLC‐Ag, this should not be considered as second‐line treatment as this was initiated shortly after TLC‐Ag application. We also demonstrated similar time‐to‐closure (67.5 days) with TLC‐NOSF compared to their study (70.2 days) with first‐line treatment with TLC‐NOSF.^27^

The Venous leg Ulcer Study IV (VenUS IV) demonstrated that the use of 2LB hosiery is as effective as 4LB with a median time of healing of 99 days with 2LB hosiery (compared to 98 days with 4LB), and similar proportion of ulcers healing (*n* = 163/230, 71%) compared to 4LB (*n* = 157/223, 70%) during a follow‐up period of up to 12 months.^5^ Hence, 2LB (UrgoK2® or UrgoK2 Lite®) was used as part of our sequential therapy. Our sequential protocol with 2LB and TLC‐NOSF showed 1 month VLU WAR of 31.2%; this is comparable to a large multicenter retrospective study of 777 patients who received compression therapy for VLU, which reported an overall monthly WAR of 30%.^33^

Compliance and tolerance to treatment is another important consideration in the management of chronic wound. While recent evidence showed that 2LB has similar healing outcomes compared to 4LB,^11^, ^12^ other advantages of 2LB over 4LB have been described. 2LB has been reported to be more tolerable as it is less bulky.^11^, ^12^ To add on, pain has been reported to be the most important factor impairing the QOL in patients with VLU.^34^, ^35^ A multicenter RCT of 187 patients comparing 2–4LB reported more significant reduction in pain over time between dressing changes when 2LB was used compared to 4LB, although this did not reach statistical significance.^36^ Our study is unable to validate this study as this is a single‐arm study assessing the efficacy and safety of pilot sequential therapy. However, we did demonstrate significant reduction in pain and discomfort after the sequential therapy (score 2.25 ± 0.78 vs. 1.63 ± 0.62, *p* = 0.003) in patients with healed VLU. Ensuring tolerability and reducing pain is also imperative for patients to be adherent to therapy and follow‐up at clinics which can improve healing rates. A retrospective study over 5 years of 155 patients with 400 venous ulcers found that guideline‐directed venous ulcer care, consisting of compression bandaging, wound dressing, and debridement, was associated with greater venous ulcer healing rates if provided at >80% of clinic visits (risk ratio: 2.52, 95% CI: 1.53–4.16).^37^ While we had 17 patients (37.8%) who were lost to follow‐up, the reasons for follow‐up were not collected and, therefore, could not be explored. It is possible that patients had significant pain or mobility difficulties with VLU resulting in default of visits, which will overestimate overall WAR. However, our results on WAR is consistent with existing literature and it is possible that patients may have defaulted clinic visits due to healed VLU, which may instead underestimate overall WAR.

Impairment of QOL is a significant complication of VLUs.^6^ Hence, we included health‐related QOL measures as our secondary outcomes, that is, the incidence of perfect health state (11,111) measured using the EQ‐5D‐5L, utility score (calculated using the US value sets), and EQ‐VAS score. The EQ‐5D‐5L has been validated to discriminate between healed and nonhealed VLUs (healed: mean 0.89, nonhealed: mean 0.73, effect size 0.76 [medium]).^23^ Our study similarly showed higher utility scores in healed VLUs (healed: 0.870 ± 0.169 vs. nonhealed: 0.812 ± 0.189, *p* = 0.39). This likely did not reach statistical significance due to our small sample size. Nevertheless, we showed improvement in QOL in patients with healed VLU, as represented by increase in incidence of perfect health states, EQ‐VAS score, and utility score.

Our study has its strengths. It is a prospective study with well‐defined inclusion and exclusion criteria and study protocol. We also included multiple outcome measures ranging from WAR to QOL measures. Our study showed comparable outcomes with individual studies reporting on the efficacy of TLC‐NOSF (compared to neutral TLC),^14^ and TLC‐Ag (compared to neutral TLC).^13^ To our knowledge, this is also the first study that reported sequential therapy with TLC‐Ag for the initial reduction of bacterial load, followed by combination of 2LB with TLC‐NOSF. Our study is limited by a small sample size of 28 which may not be representative of the VLU population locally; however, a sample size of 12 has been determined to be adequate for a pilot study.^38^ Our recruited patients also had existing VLUs (ranging 1–12 months) before the start of this study and may have received prior treatment with other wound dressings. Interpretation of our study results is also limited due to the lack of a comparator group (e.g., 2LB only or neutral TLC only). Nevertheless, the aim of this study was mainly to explore the feasibility and wound healing outcomes following the implementation of our sequential therapy rather than to compare with existing management for VLUs. We also had 17 patients who were lost to follow‐up (37.8%) who were excluded from this analysis. Reasons for follow‐up loss were not collected: patients may have had healed VLUs, which may underestimate our WAR obtained, or have adverse events or pain which resulted in defaulting subsequent visits, and hence overestimate our QOL measures. Furthermore, the PUSH scoring of VLUs were conducted manually by visual inspection of the wound and interobserver bias may be present; however, this was mitigated by having a dedicated research coordinator review the same patient as much as possible.

## 5 CONCLUSION

This prospective study demonstrated that sequential therapy for VLUs with 2 weeks of TLC‐Ag followed by TLC‐NOSF and 2LB is feasible and efficacious with good wound healing and QOL improvement in our cohort. This study should serve as a precedence for future large multi‐center well‐designed RCTs to determine the effectiveness and long‐term outcomes of sequential therapy compared to other treatment options such as TLC‐Ag alone or 2LB alone.

## AUTHOR CONTRIBUTIONS

**Natalie Shi Qi Wong**: Data curation; formal analysis; software; writing—original draft; writing—review and editing. **Audrey Hui Min Tan**: Investigation; methodology; validation; visualization; writing—review and editing. **Kai Siang Chan**: Data curation; formal analysis; software; writing—original draft; writing—review and editing. **Karine C. C. GOH**: Investigation; methodology; validation; visualization; writing—review and editing. **Peiting Lai**: Investigation; methodology; validation; visualization; writing—review and editing. **Sivakami Muthuveerappa**: Investigation; methodology; validation; visualization; writing—review and editing. **Mohamed Maliki Bin Mohamed Nasir**: Investigation; methodology; validation; visualization; writing—review and editing. **Shanying Liang**: Investigation; methodology; validation; visualization; writing—review and editing. **Qiantai Hong**: Conceptualization; formal analysis; funding acquisition; resources; supervision; writing—original draft; writing—review and editing. **Enming Yong**: Conceptualization; formal analysis; funding acquisition; resources; supervision; writing—original draft; writing—review and editing. **Zhiwen Joseph Lo**: Conceptualization; formal analysis; funding acquisition; resources; supervision; writing—original draft; writing—review and editing. All authors have read and approved the final version of the manuscript.

## CONFLICT OF INTEREST STATEMENT

The authors declare no conflict of interest.

## TRANSPARENCY STATEMENT

The lead author Zhiwen Joseph Lo affirms that this manuscript is an honest, accurate, and transparent account of the study being reported; that no important aspects of the study have been omitted; and that any discrepancies from the study as planned (and, if relevant, registered) have been explained.

## Data Availability

The authors confirm that the data supporting the findings of this study are available within the article and its Supporting Information. All authors have read and approved the final version of the manuscript. The corresponding author (Zhiwen Joseph Lo) had full access to all of the data in this study and takes complete responsibility for the integrity of the data and the accuracy of the data analysis.
